# Modernising the regulation of medical migration: moving from national monopolies to international markets

**DOI:** 10.1186/1472-6939-13-26

**Published:** 2012-10-05

**Authors:** Richard J Epstein, Stephen D Epstein

**Affiliations:** 1 Conjoint Professor, Faculty of Medicine, University of New South Wales, St Vincent's Hospital, Victoria St, Darlinghurst, Sydney, 2010, Australia; 2 Senior Counsel, Nigel Bowen Chambers, Phillip St, Sydney, 2000, Australia

**Keywords:** Registration, Medical councils, Globalisation, Bureaucracy, CME/CPD, Revalidation

## Abstract

**Background:**

Traditional top-down national regulation of internationally mobile doctors and nurses is fast being rendered obsolete by the speed of globalisation and digitisation. Here we propose a bottom-up system in which responsibility for hiring and accrediting overseas staff begins to be shared by medical employers, managers, and insurers.

**Discussion:**

In this model, professional Boards would retain authority for disciplinary proceedings in response to local complaints, but would lose their present power of veto over foreign practitioners recruited by employers who have independently evaluated and approved such candidates' ability. Evaluations of this kind could be facilitated by globally accessible National Registers of professional work and conduct. A decentralised system of this kind could also dispense with time-consuming national oversight of continuing professional education and license revalidation, which tasks could be replaced over time by tighter institutional audit supported by stronger powers to terminate underperforming employees.

**Summary:**

Market forces based on the reputation (and, hence, financial and political viability) of employers and institutions could continue to ensure patient safety in the future, while at the same time improving both national system efficiency and international professional mobility.

## Background

Rates of international medical migration have risen sharply in recent decades, creating new debates over professional opportunity and fairness [1,2]. These debates have in turn raised questions as to the utility of current professional regulatory systems, which have evolved at a relatively slow pace over the last three centuries [3]. The key challenge for any such regulatory system has always been that of protecting the public from the exaggerated claims of mere ‘druggists’ and ‘apothecaries’ [4] whilst also preventing exploitation, albeit inadvertent, by professional organisations - including those administrative bodies entrusted with regulatory powers [5]. With respect to such regulatory bodies, one factor predisposing to exploitation in the modern age is the substantial income receivable which, once entrenched as a standard requirement, can easily become self-perpetuating. Indeed, annual running costs for national regulatory offices may exceed US$100m [6].

Predictably, national Councils empowered to control physician/nurse licensing have become targets for criticism themselves. The power of the media has ensured that public perception of errors licensing foreign-trained practitioners causes severe damage, contributing to a defensive and inefficient regulatory culture [7,8]. It is therefore not surprising that pent-up disillusion with the global regulatory culture has begun to run deep in some quarters:

*The process is out of control, and quite uncaring. (Medical Councils) are seemingly above the law, do not observe ‘rules of evidence’, and are composed of people who are not doctors (and hence) have no understanding. Many careers and lives have been blighted by (their) protracted pseudo-legal activities (and) institutional hypocrisy. Medical professionals are the only group… who are assumed, often on the basis of unsubstantiated complaints, to be guilty liars, and prematurely punished [*[9]*]*.

In the light of such sentiments, we examine here the development of today’s medical licensing systems, and discuss factors implicating a need for urgent change.

### Globalisation vs. bureaucracy

Human society is changing faster than ever, driven by revolutionary increases in personal mobility (globalisation) and information transfer (digitisation). This abrupt transition to a flatter world has eroded the once-impenetrable barriers defining nation-states [10]: for example, English has become the undisputed *lingua franca* of scientific communication [11], while currencies and finance have become progressively deregulated [12]. In the medical world, international clinical services (‘medical tourism’) are now commonplace [13], north–south health dialogues are multiplying [14], practice standards are converging to global accreditation norms [15], while even the training of junior doctors [16] and nurses [17] is venturing offshore. Partly as a result of such changes, the traditional role of national agencies regulating the health sector is receiving fresh scrutiny [18]. For example, the medical licensing industry - which has long proclaimed patient safety as its sole *raison d’être*, but in practice also protects the jobs of its local clinical and administrative workforces - is now having to face such reappraisals [19]. The emerging clash is a familiar one of the new and the old: namely, in this context, the irresistible force of globalisation versus the immovable object of bureaucracy.

**Figure 1:**
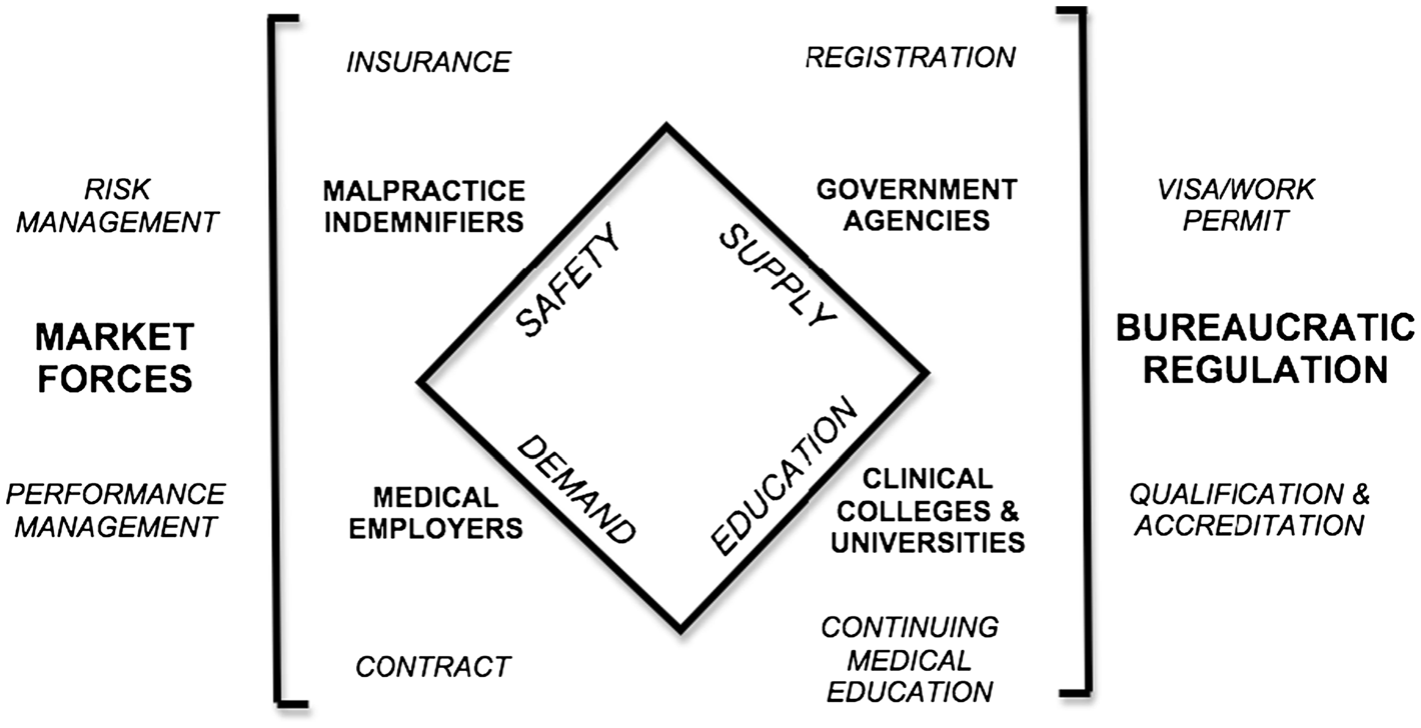
**Divergent models of regulating medical competence: performance-based (market-regulated) vs. rules-based (top-down) systems. **The market-based model, shown at left, proposes (i) to regulate medical workforce numbers through demand (i.e., the effects of institutional reputation and performance management on attracting patients, as shown at bottom left) for agreed evidence-based and cost-effective treatments, rather than through restricting provider supply as at present (top right); and (ii) to maintain the safety and quality and quality of medical work through employer- and/or insurer-led audit of relevant clinical indicators (top left) rather than relying upon adherence to centrally-administered educational programmes as currently practised (bottom right).

Bureaucracy is essential to developed societies, regulating as it does the complex activities of large populations. Yet despite its virtues, bureaucracy is negatively identified with a proliferation of 'red tape’ by many audiences - including younger people [20], businesses [21], and academics [22] –and is often criticised by specific biomedical sectors, such as those involving laboratory research [23,24], clinical trials [25,26] and ethics committees [27,28]. Indeed, some critics have even suggested the idea of ‘making bureaucracy work’ to be oxymoronic [29]. Others have noted that bureaucratic cultures are inherently fearful of debate and innovation, yet concede that rapid change can eventually force adaptations [19]. In the following sections we discuss the limitations of nation-based systems for regulating medical migration in a newly hypemobile world, and argue for a more flexible model in which market forces contribute to the regulatory framework (Figure 1).

### The Certificate of Good Standing: time to sit down

Historically, the model under which the professions operated involved conferral of professional status upon a practitioner following completion of a prescribed course of study and practice. Once conferred, the assumption was that professional status was indefinite; no assumption was made that professional skills, once learned, would atrophy, warranting removal of professional status. Hence, the original role of the regulatory authorities was itself regulated in terms of scope. This limited governance model has since been subverted by inexorably rising bureaucratic intrusion into issues of professional competence, even though such encroachment into professional spheres has seldom if ever been justified by empirical evidence. On the other hand, any such expansion of bureaucratic remit might well seem attractive to regulators, given its implications for winning more public funding and control.

Despite this recent expansion of regulatory authority in most countries, Council-centric monopolies on medical credentialing are now being pressured by an acceleration of professional migration [30]. A linchpin of international cross-credentialing in this context has been the Certificate of Good Standing (CGS) or its local equivalent, provision of which signifies ‘no complaints received by the outgoing Board’. Being a complaints-based system, individual practitioners usually have no difficulty securing a CGS in the absence of a formal allegation of misconduct against them, even if less formal complaints have been trumpeted in the press or media. Yet no CGS will be issued to any doctor against whom a complaint is pending, regardless of expected delays in considering the complaint - even if the complaint in question is patently frivolous or vexatious, or if the complaint is of a local nature that is irrelevant to the jurisdiction of the receiving Council. Indeed, if a complaint has been received but not yet considered by a Council – a process that may sometimes take months or even years to complete – the only help that will be considered by most Councils is to issue a so-called ‘Certificate of Standing’ which, by confirming that ‘Good Standing’ is not awarded, effectively confirms ‘Bad Standing’ and thus debars the practitioner - typically still licensed and practising without constraint or stigma in his/her own jurisdiction - from employment abroad. The justification of 'defending the public interest’, as is customarily invoked by regulators in this context, often exacts a heavy price on the medical needs of patients and employers abroad, not to mention the reputation and careers of practitioners seeking to migrate - who, in the majority of cases, are ultimately cleared of wrongdoing [31]. The present CGS-based system may thus offer the worst of both worlds: on the one hand, it is a potential impediment to the global mobility of well-qualified practitioners; while on the other, it offers employers little solid assurance of having excluded unsafe workers, and could even foster an illusion that all CGS-approved medical immigrants are equally competent.

### Phantom of the AHPRA

The recent history of regulation in Australia illustrates the difficulties many countries are experiencing as national health bureaucracies continue to expand. A Council of Australian Governments (COAG) agreement in 2006 led to the establishment of a national scheme for registration and training of the health workforce following recommendations made by the Productivity Committee examining supply and demand in the health workforce. Ironically, given what followed, the mandate for the COAG agreement was based on the simplification of processes that a national scheme was expected to bring.

The result was not a more streamlined system, as it turned out, but an enlarged bureaucracy - the Australian Health Practitioner Regulation Agency (AHPRA) - with redoubled authority to regulate the health workforce. To this end AHPRA was empowered to create *de novo* statutory standards for the healthcare professions, which it has so far done in relation to continuing professional development (CPD) and recency of practice. Yet by early 2011 a Senate Inquiry had been launched into the ‘chaos’, ‘massive delays’ and ‘bunglings’ of AHPRA, which had erroneously blocked registration renewals for thousands of medical practitioners; the shadow Minister for Health called the situation ‘the height of incompetence’, while even the conservative Australian Medical Association condemned AHPRA’s debut as a ‘debacle’ [32].

In hindsight, such errors are unsurprising. There could hardly have been more conducive circumstances to a fiasco than those relating to this new national scheme which overnight brought into existence an expanded unelected bureaucracy with massive discretionary powers. AHPRA provided little justification for its failings in its evidence to the Senate Inquiry, and the anonymous officials responsible for the affair never defended their actions; indeed, AHPRA couched its responses in self-congratulatory terms which denied that anything very much had gone wrong at all (AHPRA Media Release 24 March 2011; AHPRA Submission to the Senate Inquiry 14 April 2011). Such behaviour is typical of a Weberian bureaucracy, which maximises its power by the application of rules with little regard to the justification for which such rules were originally designed [33].

### The ‘brain drain’

A further example of the inflexibility of the present regulatory system relates to the net emigration of physicians from first-world countries between 1960 and 1980, partly in response to disillusionment with stifling overregulation [34]; a bureaucratic readjustment eventually reversing this trend occurred a decade later [35]. A similar professional exodus has since occurred amongst South African physicians for political reasons, and among Filipino nurses for economic reasons [36], illustrating the fluidity with which healthcare workforce skills may be transferred across borders in the globalising age [37].

It is not surprising that such high professional mobility also has its problems. For example, migrant health professionals remain vulnerable to sudden reversals of regulations affecting their employability [38,39]; highly-trained staff often face official barriers in gaining employment at anywhere near their pre-migration skills level, often ending up in relatively menial or junior roles [40]; while oscillations of national workforce oversupply and shortage are further complicated by migration fluxes [41,42]. The latter issue is politically delicate, involving as it often does the movement of health professionals from poorer to richer countries, leaving the former more in need [43]. At first glance this latter problem would appear to create a moral imperative for developed-world regulators to restrict the registration of incoming health-care workers. But the reality is more complex [44]; some developing countries have developed a successful industry in training and exporting nurses, for example, generating a valuable influx of foreign capital back to the national economy [36]. The ethics of healthcare worker migration thus need to be considered not only in medical skills terms, but also against the broader socioeconomic background [45].

## Discussion

To expedite the safe yet efficient migration of foreign (-resident or -qualified) healthcare workers, an internationally accessible database of current work-related complaints and past (upheld) offences is one quality control measure which could be created. A national Register for this purpose could be accessed by potential foreign employers on being granted permission by the job candidate in question; in support of this proposal’s credibility, it has been noted that a significant proportion of malpractising health practitioners are repeat offenders [46]. If a practitioner in country A has an unresolved complaint against his/her name, an employer in country B would have options either (i) to delay, or decide against, granting a contract, (ii) to offer a contract in which continuation was conditional upon dismissal of the complaint, or (iii) having been given the details of the complaint, to consider the relevance in country B of the complaint lodged in country A (e.g., the Hong Kong Medical Council has not allowed medical practitioners to quote Ph.D. degrees without going through a formal Council pre-approval process, whereas most other national Councils have no objection to this).

Representatives of Royal Colleges and other professional bodies have long argued the need for credentialing to be the responsibility of clinicians rather than of government. In the light of this debate, it is easy to understand the desire of both parties to control CPD requirements and/or license revalidation. Yet the perennial issues surrounding CPD - not only who should regulate it, but also how much should be carried out over what period, how it should be assessed, whether it should be linked to license renewal, and (above all) whether it actually achieves its stated justification of substantively improving patient safety - could be simplified by an employer-led model of contracts and liability. Such a system would fit the common purpose of employer and employee to maintain competence and safety; the benchmarks by which employers and employees monitor practice could be mutually agreed, strengthening audit via emphasis on meaningful clinical indicators rather than on educational marking schemes of presumed but unproven value, Indeed, to the best of our knowledge, the value of post-licensing education has never been proven in any context; for example, post-licence driver education has been reported to be ineffective in reducing car accidents [47]. These and other suggestions for change to the *status quo* are summarised in Table 1.

**Table 1:** Revised functions of national medical Boards in a market-based regulatory system

**Ongoing responsibilities of national regulatory Boards within a market-based system**	**Terminated powers of national regulatory Boards within a market-based system**
Recognition of the validity of training or experience represented by degrees or qualifications conferred locally or elsewhere	Prevention of well-qualified candidates accepting job offers agreeable to informed local medical employers and insurers
Maintaining a national register of qualified practitioners, who pay a nominal initial fee (only) for that service	Charging practitioners high annual fees solely in return for official permission to continue practising
Investigation of complaints involving professional misconduct, with the power to suspend or disqualify a practitioner from registered status if guilt is proven beyond reasonable doubt	Blocking registration for well-qualified practitioners with no track record of proven misconduct, for no reason other than that a filed complaint has not yet been evaluated by another Board
Developing mechanisms to ensure that practitioners do not over-service the patient community to an extent that is cost-ineffective, e.g. by making unfounded claims or otherwise creating excess demand	Making continued professional practice contingent upon costly and time-consuming compliance with prescribed educational activities of assumed but unproven relation to medical competence or public safety
Building transparent bridges with international regulatory partners by developing accessible online databases of complaints and disciplinary procedural outcomes	Invoking notions of privacy and confidentiality, in any setting, as a means of maintaining opacity and non-accountability, whether to the profession itself or to the public

## Summary

Bureaucracies have important benefits when first applied, such as the imposition of rule-based systems and employee protection. Such benefits tend to atrophy over time, however, being replaced by an expansionary culture that is often only curtailed by the inefficiencies and economic strains that result. Indeed, many countries are already moving their public medical systems away from bureaucracy-dominated models, and towards more market-sensitive cultures working together with clinical governance [48]. We submit that an optimal compromise between the recognised societal shortcomings of market-based systems [49] and the logistic failings of bureaucracy-dominated systems can be best maintained in the healthcare sector, as in other sectors, by a more dynamic balance of supply-and-demand forces on the one hand and electorally-guided legal reforms on the other [50].

## Abbreviations

AHPRA: Australian Health Practitioner Regulatory Agency; CGS: Certificate of Good Standing; CME: Continuing medical education; COAG: Council of Australian Governments; CPD: Continuing professional development.

## Competing interests

The authors declare no competing interests.

## Authors’ contributions

The article was conceived and approved by both authors. RJE contributed most to the medical sections, while SDE contributed most to the discussion of medicolegal issues. Both authors read and approved the final manuscript.

## Authors’ information

The views of the authors represent their own personal opinions, and not the view of any organisation with which they are associated.

## Pre-publication history

The pre-publication history for this paper can be accessed here:

http://www.biomedcentral.com/1472-6939/13/26/prepub
